# Behavioral sciences applied to acute care teams: a research agenda for the years ahead by a European research network

**DOI:** 10.1186/s12913-024-10555-6

**Published:** 2024-01-13

**Authors:** Sandra Keller, Judith G. M. Jelsma, Franziska Tschan, Nick Sevdalis, Ruth M. Löllgen, Johan Creutzfeldt, Lauren R. Kennedy-Metz, Walter Eppich, Norbert K. Semmer, Isabelle Van Herzeele, Karin Pukk Härenstam, Martine C. de Bruijne

**Affiliations:** 1 Department of Visceral Surgery and Medicine, Bern University Hospital, Bern, Switzerland; 2 Department for BioMedical Research (DBMR), Bern University, Bern, Switzerland; 3 Department of Public and Occupational Health, Amsterdam Public Health Research Institute, Amsterdam UMC, Vrije Universiteit Amsterdam, Amsterdam, The Netherlands; 4 Institute for Work and Organizational Psychology, University of Neuchâtel, Neuchâtel, Switzerland; 5 Centre for Implementation Science, Health Service and Population Research Department, KCL, London, UK; 6 Pediatric Emergency Department, Astrid Lindgrens Children’s Hospital; Karolinska University Hospital, Stockholm, Sweden; 7 Department of Women’s and Children’s Health, Karolinska Institute, Stockholm, Sweden; 8 Department of Clinical Science, Intervention and Technology (CLINTEC), Karolinska Institutet, Stockholm, Sweden; 9 Center for Advanced Medical Simulation and Training, (CAMST), Karolinska University Hospital and Karolinska Institutet, Stockholm, Sweden; 10 Department of Surgery, Harvard Medical School, Boston, MA USA; 11 Division of Cardiac Surgery, VA Boston Healthcare System, Boston, MA USA; 12 Psychology Department, Roanoke College, Salem, VA USA; 13 Department of Medical Education & Collaborative Practice Centre, University of Melbourne, Melbourne, Australia; 14 Department of Work Psychology, University of Bern, Bern, Switzerland; 15 Department of Thoracic and Vascular Surgery, Ghent University Hospital, Ghent, Belgium; 16 Department of Learning, Informatics, Management and Ethics, Karolinska Institutet, Stockholm, Sweden

**Keywords:** Research agenda, Teamwork, Acute care, Surgery

## Abstract

**Background:**

Multi-disciplinary behavioral research on acute care teams has focused on understanding how teams work and on identifying behaviors characteristic of efficient and effective team performance. We aimed to define important knowledge gaps and establish a research agenda for the years ahead of prioritized research questions in this field of applied health research.

**Methods:**

In the first step, high-priority research questions were generated by a small highly specialized group of 29 experts in the field, recruited from the multinational and multidisciplinary “Behavioral Sciences applied to Acute care teams and Surgery (BSAS)” research network – a cross-European, interdisciplinary network of researchers from social sciences as well as from the medical field committed to understanding the role of behavioral sciences in the context of acute care teams. A consolidated list of 59 research questions was established. In the second step, 19 experts attending the 2020 BSAS annual conference quantitatively rated the importance of each research question based on four criteria – usefulness, answerability, effectiveness, and translation into practice. In the third step, during half a day of the BSAS conference, the same group of 19 experts discussed the prioritization of the research questions in three online focus group meetings and established recommendations.

**Results:**

Research priorities identified were categorized into six topics: (1) interventions to improve team process; (2) dealing with and implementing new technologies; (3) understanding and measuring team processes; (4) organizational aspects impacting teamwork; (5) training and health professions education; and (6) organizational and patient safety culture in the healthcare domain. Experts rated the first three topics as particularly relevant in terms of research priorities; the focus groups identified specific research needs within each topic.

**Conclusions:**

Based on research priorities within the BSAS community and the broader field of applied health sciences identified through this work, we advocate for the prioritization for funding in these areas.

**Supplementary Information:**

The online version contains supplementary material available at 10.1186/s12913-024-10555-6.

## Background

In the United States (US), a recent study estimated that 400,000 patients admitted to the hospital may die from a medical error [1] and another study estimated one million excess injuries following medical intervention [2]. Evidence suggests that the prevalence of adverse events is higher in more complex domains of care, such as surgery and intensive care, which typically require well-coordinated teamwork [2, 3]. There is also evidence that good teamwork in healthcare is related to better performance [4]. For example, more information exchange during surgical operations can protect against complications [5, 6]. However, healthcare teams’ behaviors also contribute to generating errors, adverse events and waste of resources: Lingard and colleagues showed that communication failures during operations are common and may impact team processes [7]; higher noise levels [8, 9] and lapses in discipline [10] were also predictive of patient outcomes; and numerous disruptions increase workload and stress [11] and are associated with fewer safety checks carried out during surgical operations [12] – to name just a few of the known detrimental effects.

Interventions to improve teamwork, such as crew resource management (CRM) have been implemented in various acute care settings. In intensive care units (ICU), CRM has repeatedly been found to be beneficial for error management and job satisfaction [13–15]; further intervention studies showed promising results on patient-related outcomes in trauma, surgical, and ICU settings [16–20]. New technological developments can also influence teamwork; for example, the installation of a new communication system reduced noise disturbances in the operating room (OR) while optimizing communication [21].

In the last two decades, aspects of communication, coordination and teamwork have been identified as prominent topics studied in health care [22] with a rapid rise in scientific publications related to teams and teamwork [23]. For example, taxonomies describe key behavioral aspects at the team level [24], empirical studies relate team processes to patient outcomes [4], and investigate the impact of team interventions [25]. Yet, many studies in this domain are descriptive in nature [23] and heterogeneous, producing varying results. Although teams in healthcare have become a prominent research topic, we currently have a limited understanding of the areas in which we most lack critical knowledge to develop successful interventions that enhance teamwork and/or team skills and, ultimately, increase patient safety [26].

A European community of researchers who meet annually at the Behavioral Sciences Applied to Acute Care Teams and Surgery (BSAS) conference share a keen interest in developing the knowledge base around surgical and acute care teams’ behaviors,. The BSAS community formed over 15 years ago (2006) and represents a cross-European network of about 260 scientists and clinicians from different disciplines, committed to understanding the role of behavioral sciences in the context of acute care teams, such as surgery and interventional specialties. Most of the researchers come from northern, north-western and central-western European countries and work at universities or university hospitals. The annual conference has several goals: (a) to share research findings and experiences based on evidence-based methodologies, (b) to develop capacity (i.e., new researchers coming into the field), and (c) to ultimately contribute to improved safety, quality and outcomes through the application of behavioral interventions and training.

The BSAS community identified the need to develop a prioritized research agenda in the field of acute medical care teams. Here we report the process of developing this agenda and its prioritized areas for future research. For the present research agenda, we specifically focus on acute care teams, working predominantly in hospital settings who are often under time pressure to provide short-term, potentially invasive care to patients. These include surgery, anesthesiology, intensive care medicine, trauma, obstetrical and emergency medicine teams, but excludes teams involved with longer-term care or less acute care. During this process, we asked for suggestions over the next three to five years, implying that these issues should be tackled with more urgency, though the resulting research effort is expected to take much more time.

## Methods

The process of establishing a research agenda was initiated in 2020 by a core group (authors: MdB, JJ and SK). We used an adapted version of Zwaan and colleagues’s [27] systematic prioritization method to establish research agendas. This method weights research questions by expert prioritization criteria. The method was calibrated to draw on the expertise of the experts contacted as part of and participating in the BSAS meetings.

Using the communication channel established for the BSAS 2020 annual conference preparations, we recruited research experts for participation in establishing the list of research questions in September 2020. For establishing the prioritization weight and the assessment of the research questions according to the prioritization criteria, we collected data during the BSAS conference held virtually in October 2020; this included a half-day discussion session. Data collection was done using the Qualtrics survey software [28] and the focus groups worked with a Trello® interface [29]. In 2020 and 2021, the BSAS conference was organized virtually given the COVID pandemic and was free of charge.

### Identifying research topics

**Fig. 1:**
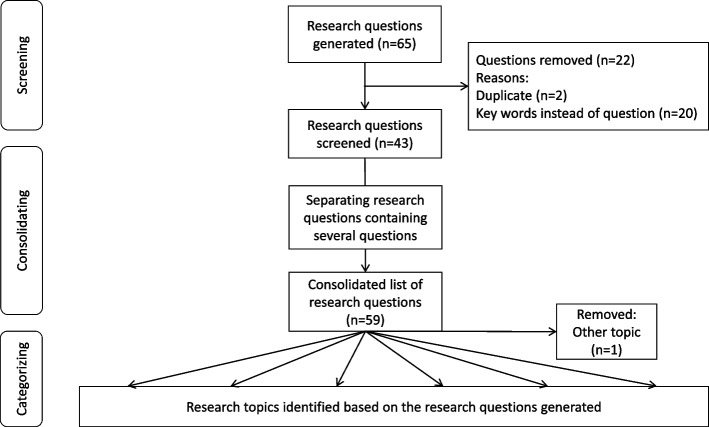
Flow diagram of the processing of the research questions generated by the experts

To identify the research topics (see Fig. 1), experts were asked to generate a list of specific research questions they considered to be the most burning for the next three to five years. Experts were recruited via the invitation to the BSAS conference 2020, including 240 researchers from the organizers’ mailing list. A total of 29 experts (12%) from different disciplines (physicians, nurses, psychologists, other) working in different settings (academic university department, surgery, anesthesiology, emergency medicine, and other fields) agreed to generate research questions. Twenty-four of the participants were active researchers, 10 were active in medical practice, and 16 had teaching assignments (multiple categories possible). A list of 65 research questions was generated.

Before categorization, the initial list of research questions submitted by the experts was consolidated by removing duplicates and entries that were too generic to be further analyzed (i.e. only single keyword, such as ‘teamwork’), as well as by separating entries with multiple research questions into several questions; one question was removed because it did not refer to behavioral research.

The resulting 59 unique research questions were categorized by two of the authors (JJ and SK) into six broader research topics. Disagreements between JJ and SK were resolved after discussion with MdB until consensus was reached.

### Prioritization criteria and prioritization of research topics

#### Prioritization criteria

To establish priorities for each research question, all participants of the BSAS 2020 online conference were invited to assess the importance of four general criteria for acute care team research. Nineteen of the 20–25 attendees agreed to participate. The criteria used were adapted from Zwaan and colleagues (2021). (i) The first criterion was the *usefulness* of the research question, i.e. to what extent it improves understanding and contributes to filling a gap in knowledge. (ii) The second criterion was *answerability*, i.e. to what extent it is realistic to reach the objective, given time, budget and ethical standards, and to what extent the endpoints are well defined. (iii) The third criterion was *effectiveness*, i.e. the potential to advance research and understanding of acute care teams; and (iv) the fourth criterion was *translation into practice*, i.e. the potential of the research for translation into practice, either directly or by supporting the development of tools to improve acute care teams. The fifth criterion used by Zwaan et al. (i.e. maximum potential for effect on diagnostic safety) was not relevant for our field and thus not considered [27]. To establish a prioritization weight for acute care team research, we also adapted the method of Zwaan et al., to the context in which the study was done and the timeline of the BSAS conference; rating only questions previously discussed as high priority by the experts, as performed by Zwaan et al., was not possible. The experts rated each of the four criteria on a sliding scale (i.e. a cursor to place on a line) between 0.5 (low importance) to 1.5 (high importance). We used the mean of the expert ratings for each criterion as the prioritization weight.

#### Assessing each research question along the prioritization criteria

The same expert group (*N* = 19) was asked to assess, for each research question, to what extent each of the four prioritization criteria (usefulness, answerability, effectiveness, potential for translation into practice) applied. The answering format was a Likert scale ranging from one star (low) to five stars (high). The research questions were presented within topic blocks, and topics were presented in a random order for each participant.

#### Calculating the weighted priority for research topics

In the next step, we calculated the weighted priority for each research question; we used a simplified version of Zwaan et al. (2021) [27] methods, since our study was conducted within a larger research field. The calculation was performed as follows: First we calculated the mean of the sum of the product of the assessment and the weight for all four prioritization criteria for each research question (priority weighting of each research question). Second, we calculated the mean of the priority-weighted research questions within each research topic. In addition, we used paired t-tests to calculate potential differences between the weights given to the criteria.

Repeated measures analysis of variance was performed to identify overall differences in the priority ratings across the six topics and two-tailed paired t-tests were calculated to identify specific differences across the topics. *P*-values below 0.05, were considered significant.

### Expert focus group meetings

In addition to the main data collection, we organized three expert focus groups during the last half-day session of the BSAS 2020 conference with the same group of 19 experts who assessed the questions based on the prioritization criteria. Experts were randomly assigned to one of the focus groups, which were composed of 5 to 7 participants each. The focus groups were asked to prioritize the importance of the research questions as high, medium, or low for acute care team research and to resolve differences of opinion by discussion; our goal was to collect expert opinion on the questions beyond the ratings. The focus groups were blind to the quantitative assessment of the research questions along with the prioritization criteria. Each focus group started with a different topic. Two groups provided an audio-recording of the discussion; the most important discussion points were summarized by (JJ, MdB); in the third group, SK captured field notes directly that summarized the discussion. The audio-recordings and the field notes served as a basis for the discussion; the prioritization made by the focus groups was not analyzed quantitatively, but instead was used exclusively to establish recommendations.

## Results

We first present the quantitative results for the research topics. For each research topic, we then present key existing literature and list research gaps, identified by the expert discussions.

### Research topics

The six research topics identified based on the research questions generated were: (i) *Team processe*s, which referred to research questions relating team processes to task execution (e.g. the impact of distractions on team outcomes, stress management in teams); (ii) *team interventions*, which referred to studying interventions to enhance team performance (e.g. design of effective team interventions, how to involve patients); (iii) *Training and health professions education*, which referred to research related to teaching, training needs, and design (e.g. teaching skills, maintaining the effects of training); (iv) *Use of technology*, which concerns research related to either the use of technology to improve teamwork (e.g. the benefits and risks of new technologies for teamwork) or the use of technology as part of research methods (e.g. team assessment technologies); (v) *organizational aspects*, including organization of work processes (e.g. care pathways), the design of work environment and schedule, and team composition (e.g. effects of changes in team composition); and (vi) *organizational and patient safety culture*, which included research questions concerning several aspects, such as steep hierarchical structures and just culture.

### Prioritization criteria

Mean expert ratings of the four prioritization criteria (on a scale from 0.5 to 1.5) were 1.21 (SD = 0.24) for usefulness, 1.15 (SD = 0.26) for answerability, 1.15 (SD = 0.26) for translation into practice, and 1.10 (SD = 0.24) for effectiveness. The means were used as weights. There was no significant difference across the means (see Supplementary Table 1 for the results of the statistical tests).

### Comparison of weighted research topics

**Fig. 2:**
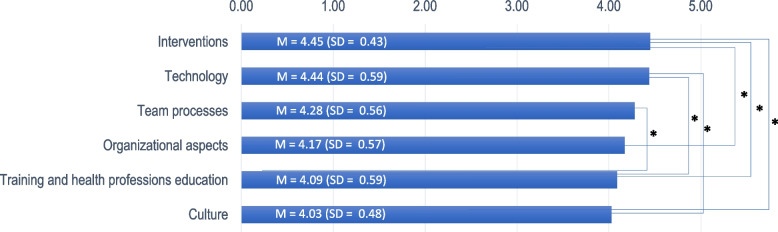
Mean, SD and significant differences across the weighted prioritization of the research topics. * *p* < .05 *Footnote below the figure *The X-axis shows the mean of the priority weighted research question (between 0 and 5) per category presented at the Y axis

Figure 2 shows the comparison of the weighted research topics in descending order. All six topics were rated as a high priority, with means above 4 (Fig. 2). ANOVA yielded significant differences between the topics (F = 4.64, df = 5, *p* = 0.023). Post-hoc comparisons revealed that the topic encompassing *interventions* was assessed as significantly higher in priority than *organizational aspects*, *training/education* and *organizational and patient safety culture*; *technology* was significantly higher than *training/education* and *organizational and patient safety culture*, and *team processes* was significantly higher than *training/education* (see Supplementary Table 2).

### Narrative results

In the following, we describe each research topic in the order of descending priority. After a general description of the topic, we present the sub-topic, based on current literature, followed by the research gaps identified during the expert focus group meetings; for a summary, see Table 1.

**Table 1:** Summary of research priorities within the six topics identified

**Topics**	**Definition of the topic**	**Sub-topics**	**Recommendations**
**Interventions to improve team processes**	Interventions that aim to improve patient care by improving team effectiveness	Interventions designed to improve: - briefings - speaking up - civility - reflexivity - patient involvement	- Focus on interventions with long-term effects - Consider research in other domains outside of healthcare
**Dealing with and implementing new technologies**	New technologies to support team interactions, including robots and the use of real-time feedback	- Impact of new technologies on team processes - Providing accurate and timely feedback	- Technologies have to be easy to use, accessible, and provide information rapidly - Take into account the local culture when implementing new technologies
**Team processes: understanding and measuring team processes**	Team processes that impact team performance, the mechanisms underlying the association and measurement of team processes and performance	- Knowledge, skills, characteristics and behaviors of teams - Validated tools to measure performance, patient outcomes, and effectiveness	- Measurement should embrace the complexity of the environment
**Organizational aspects impacting teamwork**	Inter-relatedness between work process, environment and work schedules (influences stress, sleep and work satisfaction) and team composition,	- Impact of the day, time or work shift on adverse events and outcomes - Optimization of the work environment	- Include geographically distributed teams - Support changes with optimal training
**Training and health professions education**	Learning on-the-job, simulation-based methods, principle-based training (e.g., CRM^a^) and general team training that contain multiple educational forms	Strategies to prepare teams to manage: - crisis situations - adverse conditions (e.g. rapidly changing environments, changes in team members)	- Implementation as a priority - Training as a continuous process - Tracking lasting results over time - Combining non-technical with technical skills training
**Organizational and Safety culture**	Organizations as systems that learn continuously to optimize the interaction between the work system and the worker	- Changes of patient safety over time - Association between safety culture and patient outcome	- Shift from a focus exclusively on leadership to an involvement of all team members

^a^Crew resource management

#### Interventions to improve team processes

Interventions on team processes are defined as any (organizational) intervention aimed at improving care processes through enhanced team effectiveness. The focus group discussed several sub-themes, including briefings, interventions to enhance reflexivity and encourage speaking up, promoting civility, and improving patient involvement.

The first sub-topic identified in the discussion of implementation of interventions centered around briefings. Briefings refer to specific time periods that teams dedicate to information exchange and discussion. Examples include structured patient handovers ([30, 31], Agency for Healthcare Research and [32]), or specific briefings such as the World Health Organization (WHO) or SURgical Patient Safety System (SURPASS) checklists for use in the OR [33–36]. Previous work has demonstrated that structured handovers and surgical checklists improve patient outcomes [35, 37–39]. Recently, in-action briefing interventions encouraging teams to *share information or reflect during* short task breaks have been investigated [19, 40, 41]. Teams that engage in *reflexivity*—reflecting on their goals and the team processes—have been found to be more productive [42]. Team reflexivity interventions often designate specific time slots after or between tasks for teams to review and reflect [43–47].

*Incivility* in medical teams remains a recurrent concern [48], as are *conflicts* [49, 50]; both can be a threat to patient safety. The presence of s*teep hierarchies* and *status differences* between the professions may also impede optimal interprofessional collaboration in medical teams [51, 52], potentially hindering team members from speaking up and voicing their observations, concerns, and opinions [53, 54]. *Patient involvement* [55] in this context points to patient-delivered checklists used before and after medical procedures [56], involvement of patients in checklists procedures [57] as well as patient-oriented applications designed to empower patients to contribute to their own safety whilst undergoing procedures [58].

*The primary research gaps* identified by the focus group for those topics were on the one hand to *continue research* on the relationship between team processes, interventions, and outcomes in emerging or less-explored domains such as and patient involvement. For other topics, the expert group judged that research has well established the value (e.g. briefings; ease of speaking up) or the potentially negative impact (e.g. incivility and conflict). Experts pointed out that studies from fields outside of medicine addressed these topics and should be acknowledged by scholars in the medical domain. For well-studied topics, the experts identified gaps related to implementation and strategies for effective execution. They suggested more research to compare and identify the most efficient interventional designs. Furthermore, implementation research should also explore the sustainability of the effects of interventions over time, considering that many interventional studies only include short-term effects.

#### Technology: Dealing with and implementing new technologies

New technologies are rapidly introduced in healthcare teams; they can facilitate or impair teamwork. Examples are the use of interactive whiteboards on electronic devices for collaborative decision-making of emergency cases [59], the use of artificial intelligence for OR planning tools [60], and the substitution of pagers with mobile technology [61, 62]. Notably, robotic-assisted surgery constitutes an important technological innovation, albeit presenting particular challenges for teamwork and communication [63–65], as the inclusion of a robot influences team dynamics and impacts team performance [66]. Technologies like patient portals and health monitoring wearables for patients are used to support self-management and patient engagement. Devices that gather information can facilitate shared decision making and may allow for more personalized coaching, and can expedite information sharing and exchange among team members and with patients [67]. Related to the team process, real-time data gathered from devices that continuously track team functioning indicators can provide real-time information about team performance and rapid warning signals in case of teamwork breakdown [68–70]. Additionally, the increasing integration of artificial intelligence into medical care [71–73] may extend to teamwork aspects in the future. There is limited research on the conditions and implications of current clinical practices and new technologies [74], as well as on ethical aspects related to new technology adoption [75].

The experts identified *fundamental research on the relationship between the use and impact of emerging technologies* as an important *research gap related to these topics.* They emphasized the importance of intensifying research to better understand the influence and impact of specific technologies on team processes and emergent states such as situational awareness, communication, coordination of care, team collaboration, leadership, individual and team learning processes, as well as on timely and accurate provision of performance feedback.

The experts also highlighted the necessity for research r*elated to the usability, design, and the integration of new technologies within existing clinical practice:* Inadequate system design and functionality can potentially lead to increased cognitive burden, impair clinical work, and reduce job satisfaction. Furthermore, research is needed to investigate whether technologies hamper patient privacy and psychological safety (e.g., if information is used for performance assessments or used by an insurance company). The experts stressed that the effectiveness of new technology may be moderated by the local contexts and the *organizational and patient safety culture, emphasizing that such moderators also need to be studied.*

#### Team processes: Understanding, measuring and relating team processes to outcomes

Team research for acute care teams has established solid evidence that team processes influence performance [4]. Literature reviews focused on healthcare care teams (e.g. [76]) identified similar aspects as the general teamwork literature [77], notably emphasizing team processes such as situation awareness, communication, and coordination as core non-technical skills, for example in surgery [78–81], anesthesia [82], and ICU teams [83].

Research on social and relational aspects in medical teams focused on the detrimental effects of disruptive or rude behaviors [84], on speaking up [85, 86], and on teamwork quality [87], as well as on healthcare employees’ work satisfaction and health [88].

The quantitative results showed that out of the fifteen research questions relating to team processes proposed by the experts, ten mentioned measuring performance, patient outcome or effectiveness, and six focused on how processes affect outcomes. This indicates important research gaps in relating team process to specific team task performance, including the need to develop specific indicators for medical team performance and the methodological challenges associated with performance measurement for highly complex medical tasks.

Other identified gaps were related to team composition and team diversity, specifically with regard to the optimal knowledge and skill mix of team members. Gaps were identified for both the issue of *what* the characteristics or behaviors of effective teams are and *how* diverse team processes impact performance. In addition, identified research gaps were related to contextual aspects of teamwork, including impacts of distractions and other stressful conditions at work.

#### Organizational aspects impacting teamwork

Numerous organizational aspects impact teamwork. Three topics were identified as particularly relevant by our expert group; (1) work processes, (2) work environment and work schedules, and (3) team composition.

1. Work processes: Organizational interventions (e.g. the introduction of standardized care pathways) have been shown to have positive effects on teamwork and reduce risks of burnout [89]. Research also indicates that both classroom-based team training at the department level and applying principles of a high-reliability organization (HRO) may improve job satisfaction [13] and reduce the risk of burnout [90]. However, the influence of information technology in the workplace has mainly been studied in relation to individual professional performance [91–93], whereas it may also impact teamwork in modifying or inhibiting interpersonal communication [94].
2. Work environment and schedules: Health care teams often have to provide 24/7 care and work in a context with strong hierarchies and explicit status differences [95, 96]. A strong organizational hierarchy [97] as well as inter-professional differences [98, 99] are well-known barriers to open and safe communication. The need for continuous and emergency care can only be upheld with shiftwork, which directly affects individual and team performance. Occupational safety, job satisfaction, work-life balance, and burnout are important organizational influences on teamwork [100, 101].
3. Team composition: With increasing complexity in healthcare, collaboration between multiple teams becomes increasingly important; and multiteam collaboration is a new and important research area [102, 103]. Many health care teams have low temporal stability [104] (i.e. the team composition changes daily of even for specific tasks), posing specific challenges to continuity of care as well as to the development of shared mental models and situation awareness [104].

The experts acknowledged the plethora of research in this domain, but discussed the need for research that aims to better understand how specific work environments in medicine can be optimized for functional teams. As technological innovation in health care evolves rapidly, impacting work processes (including in acute care), care is increasingly delivered by geographically dispersed teams. However, organizational aspects have mainly been studied in teams working at one location. Important research gaps pertain to the development of new theories and empirical studies on optimizing teamwork in dispersed or virtual teams or multiteam systems. In addition, the expert group identified a gap in the analyses of the impact of the work environment and schedules in terms of work shift on teamwork and outcomes.

#### Training and health professions education

Training and education of health professionals traditionally rely on an apprenticeship model of experiential learning while on-the-job with accompanying didactic approaches, including studying in the classroom and reading [105]. A rapid growth in simulation-based educational methods in the last decades aims to provide safe, effective and reproducible training [106, 107]. Simulation-based training is the most frequently investigated type of team training in medicine, followed by principle-based training (i.e. CRM and Strategies and Tools to Enhance Performance and Patient Safety (TeamSTEPPS)) [108] as well as general team trainings that contain multiple educational forms (e.g. team building, coaching, and communication skills training) [26, 109, 110].

The expert group identified research gaps for training and education that include team training under adverse conditions (e.g. over-crowded complex wards, stressful conditions, resource constraints, rapid environmental changes in demands; including evaluations of training related to specific events, for example a pandemic [111]). An important research gap relates to training for quickly changing teams, especially during crisis situations when additional people join in patient management as the crisis unfolds [112]. Furthermore, the experts emphasized that research is needed to develop training with a focus on non-technical skills that are directly connected to technical skills training, so ideally both aspects can be trained together. Another important gap is research on sustainability of training results over time and in practice, as skills learned during training are not always implemented in practice right away or at all. A proposed strategy is to provide multiple training opportunities rather than training as one-time events.

#### Organizational and patient safety culture

Current thinking about organizational and safety culture is dominated by the concept of “Just Culture” [113, 114] in relation to HROs [90], incorporating increasing complexity due to unpredictable or invisible interactions between system components and human workers. A just culture recognizes the role of the organization and its system components in providing high quality of care, and thus its responsibility in the case of adverse events, and at the same time the accountability of individual employees [115]. These aspects are emphasized in the concept of a psychosocial safety climate [116].

In order to react to disruptions and unexpected situations in a resilient manner, risks need to be managed rather than regulated [117]. Resilience research suggests that individuals and teams play an important role in managing risks and disruptions through adaptation [118, 119]. Organizations are learning systems, continually optimizing the interaction between the work system and the worker [120]. One well-known way to achieve this aim is the willingness of the organization and its employees to admit their own failures by reporting them rather than keeping them secret [121, 122]. Therefore, a “just culture” is needed, entailing an atmosphere of trust in which providers and patients are encouraged, and even rewarded, for providing essential safety-related information, but in which they are also clear about where the line must be drawn between acceptable and unacceptable behavior.

The role of leaders in influencing collective perceptions of values and priorities is frequently emphasized to establish a just culture. Psychological [123], social, and occupational safety [124] have been extensively studied as prerequisites. Leader inclusiveness, such as supporting others’ contributions, is recognized as an important determinant of team functioning and learning [125]. Theoretical understanding in this domain has grown considerably, but methods to operationalize and implement it are still in its infancy (but see Dollard and colleagues [126]).

After years of regulation and focus on leadership, there is a need for a more holistic, systemic approach, involving all team members, over a longer time frame to improve *organizational and patient safety culture*. The investigation of the relation between *organizational and patient safety culture* and patient safety outcomes was found to be of utmost importance to convince health care leaders. In accordance with these topics, questions that scored highest were related to how patient safety culture can be improved in health care organizations, as well as how to achieve a better understanding of the barriers in acute care teams to embrace team skills and strategies for inclusion of team skills in clinical curricula [127, 128].

Regarding to this topic, the expert group identified as the need to study the changes of patient safety culture over time as a research gap, as temporal changes and longitudinal studies are scarce. They also suggested to focus on studying the association between safety culture and patient outcomes more closely. Another neglected topic is research on the conditions to improve the organizational and patient safety culture. Future research should embrace a broader focus, shifting from concentrating on the role of the leader to the role of all team members.

Finally, as described in the paragraphs on themes 1 to 5, considerable interdependency exists between *organizational and patient safety culture* and team processes, technology, organization and education. For instance, organizational culture and patient safety can be strongly affected by technological and organizational structure at the hospital level and the team level. Vice versa, improvement of teamwork by tools or training can have a positive effect on organizational and patient safety culture. Furthermore, in the focus groups, local culture was discussed as a barrier to the implementation of teamwork interventions, with healthcare workers often not identifying themselves with those working outside the medical field (the “others”), and with teamwork requirements being perceived as obvious and thus undeserving of attention and resources. Research on such aspects is needed to better understand and manage these interdependencies. Proper implementation strategies, suiting the situation and context of the teams involved, should be identified for this purpose [129, 130].

### Strengths and limitations

We applied a systematic methodology to generate and prioritize research questions from a multidisciplinary group of experts in the field. Even though we likely missed important research questions (e.g. due to the low response rate to generate research questions, the participation of a limited number of mainly European experts) we believe the identified topics currently represent areas of high relevance. The circumstances of the COVID pandemic in 2020 and the fact that the conference was held virtually may have contributed to the low response rate of the experts of the BSAS community, particularly of the front-line clinicians, who were essential personnel during the pandemic. Thus, we acknowledge that the representativeness is limited by the small sample of highly specialized experts and low participation of other relevant professional groups. The adaptation of the method used by Zwaan et al. allowed us to build the research agenda with a solid community of experts in our field; however, the limitation was that experts could be involved in both the generation of the questions and their ratings, which does not align with the methods of Zwaan et al. [27]. Furthermore, even though we prioritized these research areas, we should be aware that hospitals and the broader health care setting are complex systems with many interacting parts, necessitating a more holistic or integrated approach [131]. For example, culture impacts organizational aspects, which in turn influence team processes. Consequently, the categorization of the topics performed as part of the research agenda may not reflect a more complex reality. In addition, the current research agenda primarily represents the views of experts in this field but lacks input from other relevant stakeholders such as diverse administrators (e.g. OR administrators), frontline clinicians, technology developers, and patients.

## Conclusion

We developed a research agenda with experts from the BSAS community and identified research priorities in behavioral science applied to acute care teams and surgery for the years to come. Six high-priority topics based on inputs from an expert group include: interventions; technology; team processes; organizational aspects; training and health professions education; and culture. Notably, research questions in the areas of interventions, technology, and team processes were prioritized and identified as areas where more research is needed in the near future. Interestingly, this list aligns well with the recommendations of Salas and colleagues [77] who also emphasize technology for team assessment and application among the most important future topics for teams in general. We can glean additional lessons from the research priorities identified by our group of experts, namely the urgent need to translate knowledge about impactful implementation strategies [132] effectively and sustainably.

Thus, the small and highly specialized group of experts from the BSAS network identified top research priorities in the near-term for behavioral science applied to acute care teams; these are useful for both researchers and funding agencies that operate within applied health research.

### Supplementary Information

**Additional file 1: Supplementary Table 1.** detail of the t-tests to compare the weight given by the experts to the prioritization criteria.

**Additional file 2: Supplementary Table 2.** detail of the t-tests to compare the mean ratings of the different categories of research questions.

## Data Availability

The datasets generated and analyzed during the current study are not publicly available due to the confidentiality of the research questions generated by the group of experts involved in the project, but a fully coded dataset is available from the corresponding author upon reasonable request.
