# Supplementary material for: Behavioral sciences applied to acute care teams: a research agenda for the years ahead by a European research network
Source: BMC Health Serv Res. 2024 Jan 13;24:71. doi: 10.1186/s12913-024-10555-6 (PMC10788034; doi:10.1186/s12913-024-10555-6)
Supplement: Supplementary file 1 — Additional file 1: Supplementary Table 1. detail of the t-tests to compare the weight given by the experts to the prioritization criteria. [file 12913_2024_10555_MOESM1_ESM.docx]

Supplementary Table 1: detail of the t-tests to compare the weight given by the experts to the prioritization criteria

|  |  |  |  |  |  |  |  |  |  |
| --- | --- | --- | --- | --- | --- | --- | --- | --- | --- |
|  |  | Mean Difference |  |  | 95% Confidence Interval | |  |  |  |
|  |  |  | SD | SE Mean | Lower Bound | Upper Bound | t | df | Sig.^1^ |
| Answerability with Usefulness | | -0.07 | 0.29 | 0.08 | -0.23 | 0.09 | -0.97 | 14 | 0.346 |
| Answerability with  Effectiveness | | 0.07 | 0.44 | 0.11 | -0.17 | 0.32 | 0.64 | 14 | 0.533 |
| Answerability with  Potential for Translation with | | -0.02 | 0.36 | 0.09 | -0.22 | 0.18 | -0.22 | 14 | 0.832 |
| Usefulness  Effectiveness with | | 0.14 | 0.32 | 0.08 | -0.03 | 0.30 | 1.74 | 16 | 0.101 |
| Usefulness  Potential for translation with | | 0.06 | 0.26 | 0.06 | -0.07 | 0.19 | 0.94 | 16 | 0.360 |
| Effectiveness  with potential for translation | | -0.08 | 0.28 | 0.07 | -0.22 | 0.07 | -1.11 | 16 | 0.283 |

^1^ two-sided
