# Supplementary material for: Behavioral sciences applied to acute care teams: a research agenda for the years ahead by a European research network
Source: BMC Health Serv Res. 2024 Jan 13;24:71. doi: 10.1186/s12913-024-10555-6 (PMC10788034; doi:10.1186/s12913-024-10555-6)
Supplement: Supplementary file 2 — Additional file 2: Supplementary Table 2. detail of the t-tests to compare the mean ratings of the different categories of research questions. [file 12913_2024_10555_MOESM2_ESM.docx]

Supplementary Table 2: detail of the t-tests to compare the mean ratings of the different categories of research questions

|  | | | |  | | | | | |
| --- | --- | --- | --- | --- | --- | --- | --- | --- | --- |
|  |  |  |  | | 95% confidence interval of the difference | |  |  |  |
|  | Mean | SD | SE Mean | | Lower | Upper | t | df | Sig (2-tailed) |
| Interventions with technology | 0.01 | 0.54 | 0.14 | | -0.29 | 0.31 | 0.05 | 14 | 0.961 |
| Interventions with team processes | 0.16 | 0.58 | 0.14 | | -0.15 | 0.47 | 1.11 | 15 | 0.284 |
| Interventions with organizational aspects | 0.36 | 0.62 | 0.16 | | 0.01 | 0.70 | 2.23 | 14 | 0.043 |
| Interventions with training/education | 0.35 | 0.60 | 0.15 | | 0.03 | 0.67 | 2.35 | 15 | 0.033 |
| Interventions with culture | 0.50 | 0.38 | 0.10 | | 0.28 | 0.72 | 4.93 | 13 | 0.000 |
| Technology with team processes | 0.15 | 0.49 | 0.12 | | -0.11 | 0.41 | 1.24 | 15 | 0.233 |
| Technology with organizational aspects | 0.29 | 0.54 | 0.14 | | 0.00 | 0.58 | 2.12 | 15 | 0.051 |
| Technology with training/education | 0.31 | 0.56 | 0.13 | | 0.03 | 0.59 | 2.35 | 17 | 0.031 |
| Technology with culture | 0.39 | 0.60 | 0.15 | | 0.06 | 0.72 | 2.52 | 14 | 0.024 |
| Team processes with organizational aspects | 0.18 | 0.32 | 0.08 | | 0.00 | 0.35 | 2.12 | 14 | 0.052 |
| Team processes with training and health professions | 0.22 | 0.34 | 0.08 | | 0.04 | 0.39 | 2.65 | 16 | 0.017 |
| Team processes with culture | 0.29 | 0.52 | 0.14 | | -0.01 | 0.58 | 2.07 | 13 | 0.059 |
| Organizational aspects with training/ education | -0.01 | 0.39 | 0.10 | | -0.21 | 0.20 | -0.06 | 15 | 0.951 |
| Organizational aspects with culture | 0.06 | 0.47 | 0.12 | | -0.19 | 0.32 | 0.54 | 14 | 0.600 |
| Training/education with culture | 0.08 | 0.43 | 0.11 | | -0.16 | 0.32 | 0.75 | 14 | 0.465 |
